# Electromyographic Assessment of the Masseter and Temporalis Muscles in Skeletal II Malocclusion Subjects With Varying Overjets: A Pilot Study

**DOI:** 10.7759/cureus.44645

**Published:** 2023-09-04

**Authors:** Kavitha Ramsundar, Sri Rengalakshmi, Suresh Venugopalan, Ravindra Kumar Jain, Shweta Nagesh

**Affiliations:** 1 Orthodontics and Dentofacial Orthopedics, Saveetha Dental College and Hospitals, Saveetha Institute of Medical and Technical Sciences, Chennai, IND; 2 Prosthodontics and Implantology, Saveetha Dental College and Hospitals, Saveetha Institute of Medical and Technical Sciences, Chennai, IND

**Keywords:** synergy, symmetry, surface electromyography, class ii malocclusion, class i malocclusion, muscle activity

## Abstract

Introduction

Class II malocclusions are commonly associated with some muscle disharmony and imbalance. Diagnosis of muscle imbalance helps in treating the malocclusion as well as preventing relapse of the treatment. The aim of this study is to compare the muscle activity of masseter and temporalis in patients with skeletal Class II division 1 malocclusion with varying overjet using surface electromyography (sEMG).

Materials and methods

Ten subjects in the age range 18-35 years with skeletal Class II malocclusion and varying overjets who required orthodontic treatment were included in this study. Out of these 10 patients, five of them had a 2-4mm overjet and the other five had an overjet >4mm. A four-channel sEMG system was used to conduct the sEMG of muscles. Muscle activity, synergy, and symmetry of masseter and temporalis muscles were assessed and compared between the two groups with an Independent t-test.

Results

There were no significant differences in the muscle activities of the temporalis and masseter muscles in both groups. Symmetry and synergy of these muscles in the two groups also showed no significant difference (p>0.05) at rest and clenching. However, during chewing, the masseter muscle showed poor balance and activity.

Conclusion

The overjet in Class II division 1 malocclusions did not seem to affect the muscle activity at rest and during clenching. In patients with increased overjet, during chewing, masseter activity in terms of intensity and balance was poor.

## Introduction

Angle classified malocclusions as Class I, Class II, and Class III based on sagittal dental relationships. According to Angle, Class II malocclusions are characterized as a distal relationship of the mandibular teeth relative to the maxillary teeth of more than one-half the width of the cusps [1]. According to a systematic review by Alhammadi et al., the overall global prevalence of Class II is 19.56% [2]. Class II division 1 malocclusion is associated with increased overjet while Class II division 2 malocclusion presents with retroclined incisors. Abnormal muscular patterns may be associated with either type of Class II malocclusions. Most patients with Class II malocclusion present with altered muscle and tongue activity which may contribute to the severity of malocclusion [3,4].

Electromyography (EMG) is the most common method employed to study muscle activity. It consists of electrodes connected to the skin or directly to the muscle. It detects the electrical activity of the muscle tissue and reflects it on a visual display or as an audible signal. Electromyographic indices such as symmetry (percentage overlapping coefficient) should be evaluated to include both static and dynamic operations by broadening the quantitative electromyographic analysis. This enables evaluation of the homologous, synergistic, and antagonistic muscles' function, coordination, and symmetry [5].

Muscles like the masseter, temporalis anterior and posterior, digastric anterior, and sternocleidomastoid are actively involved in functions such as chewing, swallowing, and head posture. The activity of these muscles can be examined with surface EMG (sEMG). The masseter and temporalis are typically synergists and work in tandem. Grinding and chewing functions are carried out by the masseter muscle. Meanwhile, the mandibular equilibrium and posture of the head are controlled by the temporalis muscle. Certain malocclusions can affect the masticatory functions (chewing, clenching), thereby affecting the masticatory muscles as well. It has been proposed that masticatory function and activity differ depending on the type of malocclusion (Class I, II, or III) [6]. During orthodontic treatment, modification of muscle activity may act as a stimulant [7] and aid in the treatment of muscle hyperactivity, muscle imbalance, spasm, and fatigue of the muscles of mastication [8].

The need for this study arises due to the inconclusiveness of the available literature on the association of various malocclusions with muscle activity. There is a dearth of literature on the activity of muscles like masseter and temporalis in various Class II malocclusion subjects with differing overjets. Lip biting and altered perioral equilibrium are associated with increased overjet. Studying the effect of varying overjet on muscle activity can help orthodontists understand the etiology and plan treatment, thereby preventing relapse. The aim of this study was to compare the muscle activity of masseter and temporalis in subjects with skeletal Class II division 1 malocclusion with varying overjet using EMG.

## Materials and methods

The study was prospective and was conducted in accordance with the Declaration of Helsinki guidelines and was approved by the Ethical Committee of the Saveetha Institute of Medical and Technical Sciences (SRB/SDC/ORTHO-2007/21/045). Informed consent was obtained from the participants.

Sample selection

In this pilot study, a total of 10 Class II division 1 patients equally divided into two groups (Group I - overjet 2-4mm and Group II - overjet >4mm) reporting to the orthodontic department of Saveetha Dental College, Chennai, India during January-February 2021 were screened and enrolled in the study. The inclusion criteria were as follows: patients with Class II division 1 malocclusion in the age range 18-35 and patients who have a complete set of erupted permanent teeth that are in good morphology (except third molars). The exclusion criteria were as follows: subjects who had undergone previous orthodontic treatment and subjects with craniofacial or congenital deformities, temporomandibular joint disorders, and para-functional habits.

The patients were diagnosed as skeletal Class II division 1 malocclusion based on the Sella-Nasion-Point A (SNA), Sella-Nasion-Point B (SNB), and Point A-Nasion-Point B (ANB) angles. Patients with overjet of 2-4mm were considered to be Group I and patients with patients with overjet > 4mm were categorized as Group II. Clinical examination was done with an intraoral mirror. Overjet was measured from the labial surface of the upper incisor to the labial surface of the lower incisor using a millimeter scale.

sEMG

For the apparatus, BioEMG III (BioResearch Associates Inc., Milwaukee, WI, USA) was used, and for the electrodes, four-channel electrodes were used. For electrode placement, palpation of the muscle was done, and most middle part of the muscle was identified. Anterior temporalis and masseter electrodes were placed, and the grounding electrode was positioned at the anterior medial part of the trapezius muscle.

Patient's instructions

Specific and clear instructions were given both in written and verbal format to the patients ahead of recording sEMG readings. The following directions were followed by the patients of both groups. Head and body were not to be moved during recording sEMG values, as even a slight movement might affect the output of the recording. The tongue was to be kept still and in position, as its movement would result in stimulation of other oral muscles altering the recordings of the output. A cotton ball was given to the patient and were instructed to start chewing only after a signal to start the same to clench by applying pressure.

Recording procedure

A blinded operator used a four-channel EMG system to conduct sEMG of the masseter and temporalis muscles. The sEMG is a computerized non-invasive technique utilizing an instrument that can record high-resolution sEMG patterns at 60Hz. Gelled electrodes were placed over the surface of the muscles using a self-adhesive. Muscle activities of the temporalis and masseter muscles were measured using three surface leads on each side. Of them, two were recording electrodes and the other was a reference electrode. Muscle contraction positions were palpated to determine the placement of an electrode above the temporalis muscle on both sides. The muscle contractions of the masseter were palpated by placing the fingers anterosuperior to the angle of the mandible. Here, the electrodes were positioned on both sides over the masseter muscle. The patient was reminded to follow all the instructions. A cotton ball was provided and chewing began only when the signal was issued. To make sure there was no interference with normal chewing, patients were instructed clearly to only chew on the cotton ball and to continue clenching with pressure. To begin with, the right temporalis muscle activity was allowed to rest for the first 10 seconds while being recorded. This was followed by chewing on the right side only and then resting for 10 seconds each time. The same procedure was repeated for the left temporalis muscle. The sEMG values were recorded for the left temporalis muscle first at rest, then while chewing again at rest. All three activities were recorded for 10 seconds each. Lastly, both the right and left temporalis muscles were evaluated for 10 seconds at rest, followed by simultaneous clenching on both sides for 10 seconds and the last 10 seconds at rest. Masseter and temporalis muscles were evaluated for their activity during both actions and the values were noted down individually. The results were displayed by the software and measurements adapted are measured in µV.

Statistical analysis

An independent t-test was done to compare the symmetry and synergy of muscles of both Group I and Group II groups using SPSS Statistics version 17.0 (SPSS Inc. Released 2008. SPSS Statistics for Windows, Version 17.0. Chicago: SPSS Inc.).

## Results

The study was conducted for a period of three months. A total of 10 patients were recruited for the study, of which six were males and four were females. The mean age of the patients was 22+/-2.5 years.

Muscle activity, synergy, and symmetry of the masseter and temporalis muscles were assessed. Mean symmetry and synergy values are tabulated (Tables 1-2). Intergroup comparisons for the symmetry of the right temporalis muscle (p-value 0.27), left temporalis muscle (p-value 0.89), right masseter muscle (p-value 0.09), and left masseter muscle (p-value 0.50) were not statistically significant. It can be inferred that the studied muscles had no differences in symmetry between the groups. Though the differences were not statistically significant, the mean symmetry of temporalis and masseter in Group II was higher than in Group I.

**Table 1:** Mean for symmetry of the temporalis and masseter muscle

Groups	Mean	Standard deviation	p-value
Temporalis right: Group I	124.820	57.4453	0.275
Temporalis right: Group II	158.460	28.6343	
Temporalis left: Group I	132.900	58.1737	0.895
Temporalis left: Group II	137.360	44.7121	
Masseter right: Group I	152.540	89.1826	0.094
Masseter right: Group II	230.340	21.4120	
Masseter left: Group I	189.180	128.7268	0.501
Masseter left: Group II	235.060	68.1444	

**Table 2:** Mean for synergy of the temporalis and masseter muscle

Groups	Mean	Standard deviation	p-value
Temporalis right: Group I	75.800	38.4232	0.832
Temporalis right: Group II	81.220	39.7410	
Temporalis left: Group I	76.020	46.1955	0.334
Temporalis left: Group II	53.540	15.9735	
Masseter right: Group I	138.480	196.7586	0.949
Masseter right: Group II	132.640	32.2898	
Masseter left: Group I	131.940	165.8773	0.736
Masseter left: Group II	105.880	18.1414	

Intergroup comparison of the synergy of right temporalis muscle (p-value 0.83), left temporalis muscle (p-value 0.33), right masseter muscle (p-value 0.94), and left masseter muscle (p-value 0.73). It can be inferred that the studied muscles had no differences in synergy between the groups. However, the synergy of masseter and temporalis was lower overall in Group II than in Group I.

**Figure 1:**
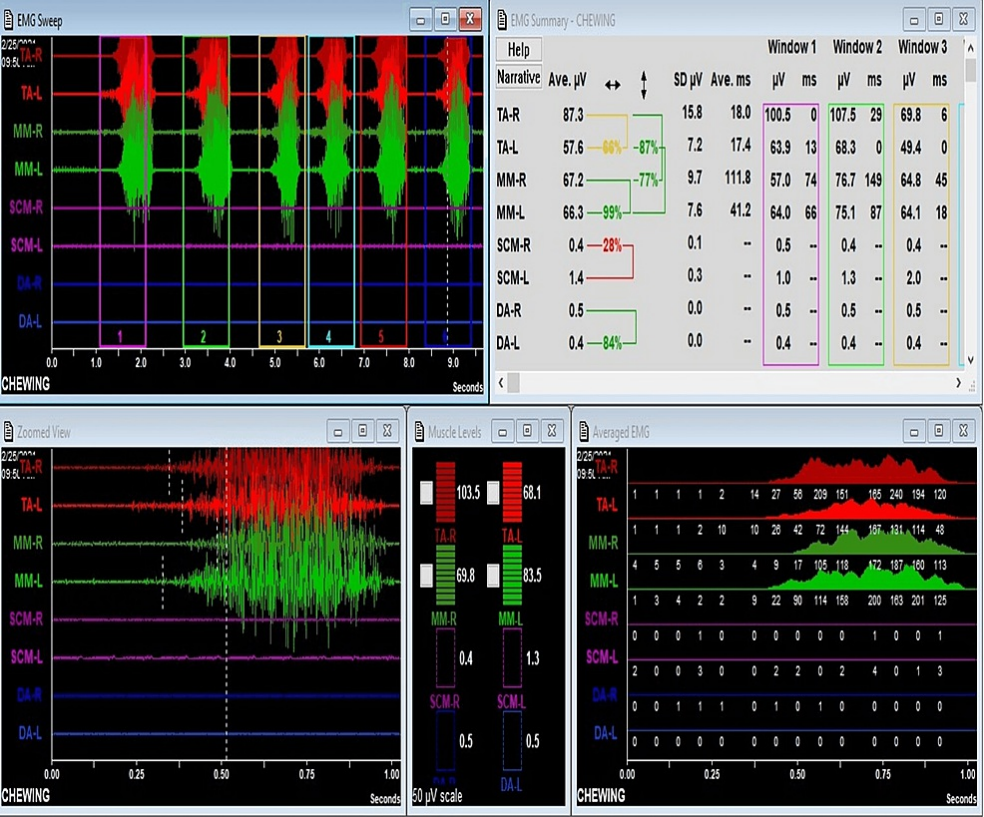
sEMG recording for Group I during chewing The sEMG recording shows the left temporalis gets activated first during chewing cycle. The balance of functioning between right and left temporals is fair (symmetry). The balance of functioning between left temporalis and left masseter is good (synergy). Masseter functioning balance between right and left is good (symmetry).

**Figure 2:**
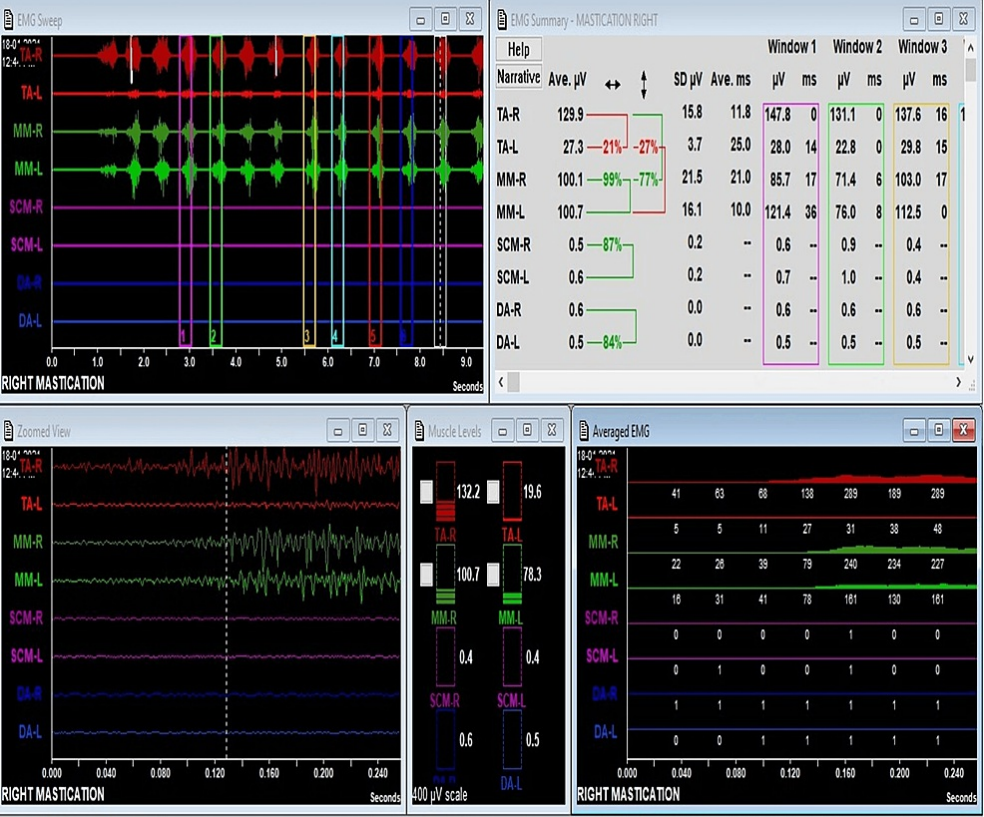
sEMG recording for Group II during chewing The sEMG recording shows that the left temporalis left gets activated first during chewing cycle. The balance of functioning between right and left temporals is poor (symmetry). The balance of functioning between left temporalis and left masseter is poor (synergy). Masseter functioning balance between right and left is good (symmetry).

The mean symmetry and synergy for the masseter and temporalis muscle during clenching and chewing are mentioned in Tables 3-4. The intensity and balance were interpreted as follows: if the values are >60%, there is good intensity and balance; 40-60%, fair intensity and balance; and <40%, poor intensity and balance. During chewing, in Group I, fair intensity and balance were noted between the right and left side muscles. During chewing, in Group II, fair intensity and balance were noted between the right and left side muscles. During clenching, in Group II, fair intensity and balance were noted between the right and left side muscles (Table 3). During chewing, in Group II, poor intensity and balance were noted between the temporalis and masseter muscles (Table 4). The sEMG recording showing during chewing is depicted for Group I and Group II in Figures 1-2, respectively.

**Table 3:** Symmetry and synergy of temporalis and masseter muscles of the same side during chewing and clenching

	Chewing		Clenching	
	Right temporalis and masseter	Left temporalis and masseter	Right temporalis and masseter	Left temporalis and masseter
Group I	40%	80%	60%	60%
Group II	80%	60%	40%	80%

**Table 4:** Synergy and symmetry of the temporalis and masseter muscles separately during chewing and clenching

	Chewing		Clenching	
	Right and left temporalis	Right and left masseter	Right and left temporalis	Right and left masseter
Group I	80%	60%	80%	60%
Group II	60%	20%	80%	60%

## Discussion

The present study recorded and compared the muscle activity in terms of symmetry and synergy of masseter and temporalis in patients with skeletal Class II division 1 malocclusion with varying overjets using sEMG. The muscle activity of both masseter and temporalis was measured at rest, chewing and clenching using sEMG which uses surface electrodes and detects superimposed motor unit action potentials from many fibers, as opposed to the single ones recorded by the intramuscular type [9]. The present study utilized sEMG instead of intramuscular EMG due to its non-invasive nature. The readings obtained are also objective and precise as per the review by Woźniak et al [5].

The symmetry of the right and left temporalis and the right and left masseter muscles was higher in subjects with overjet of more than 4mm but not significantly different; hence, it can be inferred that overjet differences did not influence muscle symmetry. Intergroup comparison of the synergy of the right and left temporalis and the right and left masseter was similar in both the studied groups of included subjects; hence, it can be inferred that overjet differences do not influence the synergy of muscles in Class 2 division I subjects. During chewing in Group I, fair intensity and balance were noted between the right and left side muscles, and in Group II, poor intensity and balance were noted between the temporalis and masseter muscles. During clenching, in Group II, fair intensity and balance were noted between the right and left side muscles.

In the study by Nishi et al. [7], the association between overjet and activity of masseter and temporalis was assessed. The study found an association between increased overjet in Class II patients and altered masseter activity during chewing. Moreno et al. [10] evaluated the electromyographic behavior of jaw muscles during clenching, swallowing, and chewing in different malocclusions. The temporalis muscle is the most affected in Class II malocclusion during swallowing and chewing which was not similar to the findings of the present study. In the present study, the temporalis muscle of Group I has good intensity and balance, and Group II showed fair intensity and balance.

Ocak et al. [11] evaluated the orbicularis oris and masseter muscle activity changes after upper incisor protrusion in Class II division 2 malocclusion. The study basically tried to evaluate if the proclination of teeth during orthodontic treatment can have any effect on muscle activity. The study concluded that the activity of orbicularis oris increased after the protrusion of upper incisors, but the activity of the left and right masseter decreased and increased back to the initial values during the retention period. A study by Thüer et al. [12] evaluated changes after protrusion and intrusion of incisors in Class II division 2 cases. The authors found that the activity of the anterior and posterior temporal muscles was decreased during protrusion and intrusion of the upper incisors, whereas the activity of the masseter muscles was not significant. The activity of temporalis in the present study is similar to these findings. The study by Ahlgren et al. [13] compared the EMG activity of masseter and temporalis in children with Class II division 1 malocclusion and Class I occlusion. The study found decreased EMG activity of the temporalis and masseter in the Class II group when compared to normal occlusion. The study evaluated only the maximum voltage and mean duration of EMG activity in both groups during chewing and swallowing, while the present study assessed symmetry and synergy.

EMG for diagnosis and treatment planning in orthodontics has been suggested for quite some time. In the clinical as well as research domain, surface EMG can aid in the diagnosis and management of muscle hypertrophy, hyperactivity and hypoactivity, estimation of rest position, and evaluation of muscular imbalance [6]. The sEMG can also be used to assess muscle activity after orthodontic treatment. Castaneda et al. reported that there were variations in the muscular activities recorded at various stages of orthodontic treatment. The authors concluded that masseter muscle activity showed variation and stabilized during the finishing stage of treatment or during the use of a physiological splint, where the occlusal contacts are found to be distributed more uniformly [14]. Pancherz et al. reported in their study that using the Herbst appliance (employing continuous bite jumping) to treat Class II division 1 malocclusion increased the EMG activity from the temporal and masseter muscles during the period just after the removal of the appliance. They stated the change in the sagittal jaw base or dental relationship most likely resulted in a shift in muscle function [15].

The assessment of muscle activity in various malocclusions and the effect of various factors like increased overjet on muscle activity is essential for understanding the etiology of the varied presentations of Class II malocclusion. This understanding can help in accurate diagnosis and treatment and also in minimizing retention.

Limitations

The main limitation of the present study is the sample size. The study was a pilot study, and, hence, a study in larger is required to generalize the results. In addition, the muscle activity was assessed at one point in time only. A longitudinal analysis of the muscular activity throughout the management of increased overjet in Class II division 1 malocclusion can be assessed to have a comprehensive knowledge of the muscular activity. The study primarily assessed the activity of masseter and temporalis. The action of orbicularis oris can also be assessed in patients with different overjet.

## Conclusions

The degree of overjet in Class II division 1 malocclusions did not seem to affect the muscle activity at rest and during clenching. In patients with increased overjet, during chewing, masseter activity in terms of intensity and balance was poor. Further studies are required to assess if there are changes in muscle activity following growth and treatment.
